# Defining apnea of prematurity is messy

**DOI:** 10.1038/s41390-025-04723-5

**Published:** 2025-12-23

**Authors:** David M. Rub, Eric C. Eichenwald, Richard J. Martin

**Affiliations:** 1 Division of Neonatology, Children’s Hospital of Philadelphia, Philadelphia, PA USA; 2 Department of Pediatrics, University of Pennsylvania Perelman School of Medicine, Philadelphia, PA USA; 3 University Hospitals Rainbow Babies & Children’s Hospital, Case Western Reserve University, Cleveland, OH USA

The occurrence of apnea in premature infants is no surprise given the limited role for respiration in utero. The prominence of apnea with accompanying bradycardia has been long recognized and Daily concluded in 1969 that ”continuous monitoring of respiration in small infants is now clinically feasible”.^1^ In that study the relatively new technique of impedance technology, which measured electrical resistance across the chest, quantified chest wall motion and heart rate. Noninvasive blood gas monitoring to identify resultant hypoxemic episodes would not be widely available for another decade. Initial concerns about the limitations of impedance monitoring in the NICU emerged in the 1990s with recognition that upper airway obstruction is a major contributor to longer apneic episodes and this would be missed by impedance monitoring.^2^ Unfortunately, the technologic challenge to directly measure ventilation in the NICU has had limited success.

The ability to accurately identify and quantify apnea in the NICU setting is important. Apnea of prematurity is an important physiologic link between the immature brain and lung, it can compromise gas exchange, and prolongs NICU hospitalization. Our inability to reliably monitor apnea via impedance has led caregivers to focus on the associated bradycardia and hypoxemia. There are limitations here too as the precise mechanism for accompanying bradycardia is not well understood and intermittent hypoxemia during apnea is contributed to by lung immaturity and even pulmonary hypertension. Nonetheless there are clearly documented associations between intermittent hypoxemia and various morbidities. These include retinopathy of prematurity,^3^ bronchopulmonary dysplasia,^4–6^ prolonged hospitalization^7^ and developmental delay.^8,9^

In this scoping review, Jeanne et al. set to advance the field by describing the heterogeneity of definitions and monitoring techniques used by investigators studying apnea of prematurity. The authors aptly note the importance of standardization to facilitate evaluation of the effects of interventions on apnea burden in premature infants. This lack of consensus in monitoring techniques and definitions is partly responsible for the fact that many studies of apnea and related intermittent hypoxia in preterm infants opt to measure their downstream clinical effects such as bronchopulmonary dysplasia or neurodevelopmental impairment rather than apnea burden itself.^10,11^ This hinders our ability to understand the true causal mechanism of therapies targeting apnea of prematurity such as caffeine or continuous positive airway pressure (CPAP).

In addition to reporting the remarkable heterogeneity of apnea definitions, the authors list the most common citations for these definitions. The definitions from those citations are summarized in Table 1. Notably, the most common citation was that of the American Academy of Sleep Medicine (AASM),^12^ likely due to 33% of the included studies not being limited to preterm infants. The AASM definition was designed to be inclusive of all children <18 years of age and to be recorded in an accredited diagnostic sleep lab with air flow (i.e., nasal thermistor or nasal pressure transducer) and respiratory effort measurement devices. In adopting a standardized definition, we think it is critical to choose a pragmatic definition that can be used reliably within the NICU for both clinical and research purposes and is specific to the pathophysiology of apnea in premature infants.

**Table 1:** Common definitions of apnea spells in premature infants.

Definition	Intended Population	Apnea Classification	Inspiratory Effort	Duration of Apnea	Heart Rate Threshold	Oxygen Saturation	Other Criteria
AASM^12^	Infants <1 year of age	Central	Absent	≥2 breaths AND	<50 bpm for 5 s or <60 bpm for 15 s OR	≥3% arterial oxygen desaturation OR	Arousal
				≥20 s^a^			
	Children <18 years of Age, including Infants	Obstructive	Present	2 breaths			
		Mixed	Both				
Summary Proceedings of AoP Group^15^	Preterm Infants <37 weeks GA	Central, Obstructive, or Mixed		10–20 s	<80 bpm	SpO2 < 80–85%	
				≥20 s			
COFN AoP Clinical Report^16^				10–20 s	<100 bpm	Cyanosis OR	Pallor
				≥20 s			
Lee et al.^13^		Central		≥20 s			
				10–20 s	<100 bpm	SpO2 < 80%	
		Central – Severe^a^		30 s			

*BPM* beats per minute, *GA* gestational age, *COFN* Committee of Fetus and Newborn, *AOP* Apnea of Prematurity, *AASM* American Academy of Sleep Medicine.

^a^Severe event definition derived from Ramanathan et al. JAMA 2001.^17^

The challenge of establishing standardized definitions is intertwined with our ability to accurately detect and classify apneas. Given the practical constraints of the NICU environment, algorithm-enhanced transthoracic impedance (TTI) monitoring represents the most promising solution for improving apnea detection. Jeanne et al. report that optimized algorithms can achieve central apnea detection sensitivities of 78–94%, a substantial improvement over conventional bedside monitoring (9–64%).^13,14^ Though technologies such as respiratory inductance plethysmography, nasal thermistors, or nasal pressure transducers increase our ability to detect obstructive events, use of these devices are likely to not be feasible in everyday practice. Visual detection technologies, leveraging improving machine learning and artificial intelligence may help fill this gap, but additional research and development is still needed in this field.

However, even with improved detection technology we are still left with the fundamental question of how much apnea or hypoxia is clinically important. Is a 15-s pause with heart rate dropping to 90 beats per minute clinically equivalent to a 10-s pause with heart rate to 70? Does the oxygen desaturation threshold of 80% versus 85% matter for long-term outcomes? These questions remain unanswered. Pragmatic studies comparing different alarm thresholds and their relationship to both immediate clinical interventions and long-term outcomes are desperately needed.

This scoping review by Jeanne and colleagues lays important groundwork by systematically documenting the mess of definitions and monitoring approaches currently used in apnea research. Their analysis clarifies what we need: standardized definitions that are both physiologically meaningful and practically measurable, coupled with research linking specific apnea characteristics to clinically important outcomes. Without the ability to reliably identify, classify, and quantify apneas in a standardized way, we cannot hope to optimize how we manage apnea of prematurity or improve outcomes for these fragile infants.
